# Differential effect of monoterpenes and flavonoids on the transcription of aromatic ring-hydroxylating dioxygenase genes in Rhodococcus opacus C1 and Rhodococcus sp. WAY2

**DOI:** 10.1099/mgen.0.001359

**Published:** 2025-03-05

**Authors:** Andrea Zubrova, Manuela Tadrosova, Jaroslav Semerad, Tomas Cajthaml, Petr Pajer, Michal Strejcek, Jachym Suman, Ondrej Uhlik

**Affiliations:** 1 Department of Biochemistry and Microbiology, Faculty of Food and Biochemical Technology, University of Chemistry and Technology, Prague, Prague, Czech Republic; 2 Institute of Microbiology, Academy of Sciences of the Czech Republic, v.v.i., Prague, Czech Republic; 3 Institute for Environmental Studies, Faculty of Science, Charles University, Prague, Czech Republic; 4 Military Health Institute, Ministry of Defence of the Czech Republic, Prague, Czech Republic

**Keywords:** aromatic pollutants, aromatic ring-hydroxylating dioxygenases, biodegradation, flavonoids, monoterpenes, *Rhodococcus*

## Abstract

Aromatic ring-hydroxylating dioxygenases (ARHDs) play a crucial role in the aerobic biodegradation of both natural and anthropogenic aromatic compounds. Although their ability to process contaminants is not entirely understood, it is thought to have evolved from the transformation of structurally similar secondary plant metabolites (SPMs). Hence, to investigate this connection, we tested a variety of SPMs from the monoterpene and flavonoid classes as carbon sources and transcriptional effectors of several phylogenetically distant ARHD genes involved in the degradation of aromatic pollutants. Specifically, we focused on *bphA1*, *nahA1* and *phtA1* in *Rhodococcus opacus* C1, whose genomic analysis is also presented hereinafter, and *bphA1a*, *nahA1-bphA1b* and *etbA1ab* in *Rhodococcu*s sp. WAY2. Whilst induction was only observed with (*R*)-carvone for *bphA1a* and *nahA1-bphA1b* of strain WAY2, and with *p*-cymene for *nahA1* and *nahA1-bphA1b* of strains C1 and WAY2, respectively, an extensive inhibition by flavonoids was observed for most of the genes in both strains. To the best of our knowledge, our study is the first to report the effect of flavonoids and monoterpenes on the transcription of *nahA1*, *etbA1* and *phtA1* genes. In addition, we show that, in contrast to pseudomonads, many flavonoids inhibit the transcription of the ARHD genes in rhodococci. Thus, our work provides a new perspective on flavonoids as the transcriptional effectors of ARHDs, highlighting the significant variability of these enzymes and the divergent responses that they elicit. Moreover, our results contribute to understanding the complex interactions between microorganisms and SPMs and provide insights into the molecular basis of a number of them.

## Data Summary

The complete assembly for *Rhodococcus opacus* C1 is available in the National Center for Biotechnology Information GenBank under the BioProject accession number PRJNA1028948. The measured quantitative PCR data are available online at https://doi.org/10.5281/zenodo.14217697. The authors confirm that all supporting data and protocols have been provided within the article or through supplementary data files. Five supplementary figures and two supplementary tables are available with the online version of this article.

**Impact Statement**

Aromatic ring-hydroxylating dioxygenases (ARHDs), which are involved in the degradation of plant metabolites, are thought to be exapted by soil bacteria to transform structurally similar pollutants. However, the exact relationship between plant metabolites and ARHDs remains unclear. Consequently, our study demonstrates that a number of flavonoids and monoterpenes significantly influence numerous ARHD genes, including some that have been underrepresented in research to date. Whilst our findings support previous studies suggesting that plant metabolites may enhance ARHD-related pollutant degradation, they also underscore the diversity of plant metabolites and ARHDs, highlighting the need for further research.

## Introduction

ARHDs (aromatic ring-hydroxylating dioxygenases) are multi-component non-heme oxidoreductases that play a key role in the aerobic degradation of aromatic compounds; they incorporate oxygen atoms, thus increasing the reactivity of an otherwise stable aromatic ring and enabling its further cleavage [1]. ARHDs provide certain bacteria with organic carbon and energy sources for growth or simply detoxify persistent aromatic compounds such as plant metabolites or pollutants. In fact, ARHDs have been mostly described as pollutant-degrading enzymes [2]. However, much has yet to be understood about their ability to transform anthropogenic pollutants, which have only been introduced into the environment in significant quantities within the last century [3,8].

One of the leading hypotheses as to why soil-borne ARHDs efficiently transform pollutants stems from the co-evolution of plants and micro-organisms, specifically the interplay between soil-borne bacterial ARHD activity and the metabolism of secondary plant metabolites (SPMs) [8]. SPMs, a diverse and plant-unique variety of compounds, many of which are based on aromatic rings, often possess antimicrobial properties, disrupt quorum sensing or act as signalling molecules in different ecological processes [9,11]. Bacteria supposedly recruit ARHDs to transform and detoxify SPMs but also thrive on new carbon sources [12,13]. Finally, due to the structural similarity of SPMs to pollutants, it has been proposed that bacteria recruit and adapt ARHDs originally involved in SPM metabolism to degrade pollutants [8]. Thus, ARHDs' involvement in pollutant degradation may result from their exaptation [14].

One example of such a pollutant-degrading ARHD involved in SPM metabolism is BphA, a large subunit (LSU) of biphenyl 2,3-dioxygenase, a key enzyme of the biphenyl pathway, which is also involved in the degradation of polychlorinated biphenyls (PCBs). Pham *et al*. reported that flavanone and ten other flavonoids acted as substrates for the BphA1 enzyme in *Rhodococcus erythropolis* U23A [15]. Furthermore, flavanone was found to act as an inducer of *bphA1*, which aligns with the previous work of Toussaint *et al.* [16]. More recently, we have also demonstrated that the *bphA* gene in *Ectopseudomonas alcaliphila* JAB1 (reclassified from the former *Pseudomonas alcaliphila* JAB1 by Rudra and Gupta [17]), a versatile PCB degrader, is induced by a broad spectrum of both phenolic compounds as well as monoterpenes. Moreover, the JAB1-borne BphA catalysed the transformation of flavone, flavanone and (*S*)-limonene [18].

Although both pseudomonads and rhodococci contribute to biphenyl/PCB transformation, the relationship among their phylogenetically distinct ARDHs, biphenyl/PCB transformation and SPMs remains to be explained. Indeed, members of the genus *Rhodococcus* are powerhouses of catabolic enzymes. Among other features, their genomes contain numerous extrachromosomal elements with multiple gene copies for a wide range of ARHDs that have been underrepresented in research to date, making rhodococci ideal model organisms among soil bacteria for study [6,19,22], especially given the fact that they thrive not only in a variety of soil types but also in marine and freshwater environments, as well as in host-associated environments such as insect guts and plant endospheres [23,25]. Given their ability to succeed in a range of challenging environments, including contaminated sites with harsh conditions, there has been considerable interest in their potential for bioremediation, as evidenced by previous studies [26,28].

Hence, in this study, a variety of SPMs of the monoterpene and flavonoid classes were investigated as carbon sources and potential inducers of multiple ARHD genes in two soil rhodococci: *bphA1*, *nahA1* and *phtA1* in *R. opacus* C1 and *bphA1*, *nahA1* and *etbA1* in *Rhodococcus* sp. WAY2. These ARHD genes are phylogenetically distinct and widen the potential abilities of rhodococci to degrade various aromatics. In our previous work, we identified several flavonoids and monoterpenes that induced *bphA* gene transcription in *E. alcaliphila* JAB1; in this work, we are showing that rhodococcal ARHD genes respond to these SPMs differently.

## Methods

### Source of micro-organisms, culture media and growing conditions

The bacterial strain *Rhodococcus opacus* C1 was isolated from soil at a wood processing facility in Ümea, Sweden (63.870493 N; 20.414548 E) on 20 February 2013. Due to the wood processing, the soil was enriched by lignocellulose and contaminated by pentachlorophenol, chlorinated dibenzofurans and dibenzodioxins. Its genome sequence was analysed using the Oxford Nanopore sequencing platform and deposited in the National Center for Biotechnology Information database under BioProject accession number PRJNA1028948. For more details, see Supplementary Material: Genome analysis of the strain C1. The strain *Rhodococcus* sp. WAY2 was isolated from a rhizosphere near a petrol station in Spain as reported by Garrido-Sanz *et al*. [29] and was kindly provided by the authors for this research. Both strains were stored in glycerol stocks (25% v/v) at −80 °C and revived on PCA (plate count agar; Oxoid, UK) plates. Further cultivation proceeded in LB (Luria Broth) medium (Sigma-Aldrich, USA) and MSS (minimal salt solution [30]). MSS was supplemented with 30 mM fumarate (Sigma-Aldrich, USA) as the sole carbon source. Liquid cultures were incubated on a rotary shaker at 28 °C, 130 r.p.m.

### Genome sequencing of strain C1 and phylogeny reconstruction of protein sequences of strains C1 and WAY2

The bacterial genome sequencing was carried out following the procedure outlined in Supplementary Material: Genome analysis of strain C1.

For the phylogeny reconstruction, aa sequences (see Supplementary material: Search for ARHDs, Table S1) deduced from the annotated whole-genome sequence of the strain C1 and WAY2 were searched via blastp against the RefSeq database [31,32]. The protein sequences of the top hits, along with an exemplary set of yet-characterized ARHDs [33], were aligned using MAFFT (G-INS-i strategy) [34]. The maximum-likelihood phylogenetic tree was reconstructed within IQ-TREE software (best fitting model LG+R4 selected via ModelFinder, nonparametric bootstrap value 1000) [35,36].

To visualize and compare the organization of the ARHD-bearing gene clusters in strains C1 and WAY2, the Clinker tool was used with the respective GenBank files [37].

### Chemicals

**Fig. 1.**
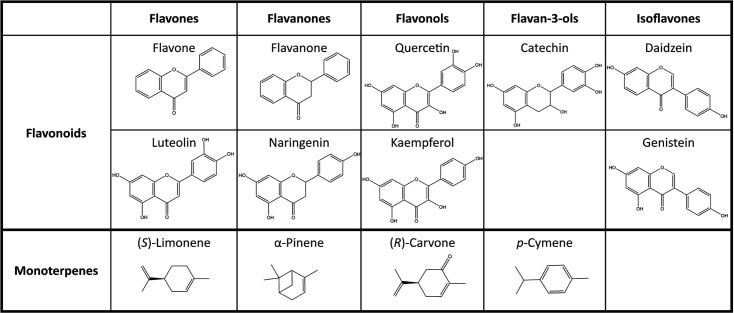
Structures of SPMs used in this study.

Stock solutions of SPMs (Sigma-Aldrich, USA) were prepared by dissolving commercial substances in appropriate solvents depending on the assay, i.e. ethanol or DMSO. The SPMs tested were flavonoids (flavone, flavanone, luteolin, daidzein, genistein, catechin, naringenin, quercetin and kaempferol) and monoterpenes [(*S*)-limonene, *α*-pinene, (*R*)-carvone and *p*-cymene)] (Fig. 1). Other compounds tested are listed in Table S2. Solutions were sterilized by microfiltration (0.22 µm) and stored in the dark at 4 °C. Biphenyl stock solution (0.1 mM) was prepared by dissolving biphenyl in ethanol freshly prior to use.

### Utilization and transformation of SPMs

In strains C1 and WAY2, the capacity to utilize SPMs was tested as follows: freshly grown cells of each strain in LB media were washed twice in physiological solution and inoculated into Erlenmeyer flasks containing MSS (1% v/v) amended with 1 mM of SPM after previous ethanol evaporation. Cell growth was monitored by OD_600_ measurement after 24 and 72 h of cultivation or by plate counts on PCA in the case of low-soluble SPMs (flavonoids).

The ability of strains C1 and WAY2 to transform SPMs was tested by performing a resting-cell assay (RCA). Briefly, freshly grown cells of each strain in LB media were harvested (10 min, 5000 ***g***) and washed twice with a physiological solution. The cell suspensions were inoculated (OD_600_ of 0.05) in Erlenmeyer flasks containing MSS amended with 30 mM fumarate and 0.5 mM biphenyl. The cultures were incubated at 28 °C, 130 r.p.m. for 48 h for strain C1 and 24 h for strain WAY2. The cells were harvested by centrifugation, washed twice with physiological solution and adjusted to an OD_600_ of 1 in MSS. The bacterial suspensions (1 ml) were added to 8 ml glass vials along with the tested SPM (dissolved in DMSO) at a final concentration of 100 µm. Simultaneously, the negative control was prepared with autoclaved bacterial cells. The assay was performed in triplicates for each SPM with both viable and autoclaved cells. Assays were cultivated for 24 h (28 °C, 120 r.p.m.). Incubation of samples containing flavonoids was terminated by 1 ml of methanol (HPLC-grade, Lach:ner, CZ). Subsequent analysis was performed on reverse-phase HPLC coupled with a UV-VIS diode array detector (Nexera XR station, Shimadzu) (for more details, see Supplementary Material: Analysis of flavonoids). The incubation of samples containing monoterpenes was terminated by freezing them at −80 °C. Further analysis was performed by GC coupled with an MS detector (for more details, see Supplementary materials: Analysis of monoterpenes). The levels of SPMs after the incubation with viable and autoclaved cells were compared using a two-sample t-test (*α*=0.05).

### Transcription of ARHD genes in strains C1 and WAY2 in the presence of SPMs and biphenyl

The effect of SPMs on the transcription of genes *bphA1*, *nahA1* in strain C1 and *bphA1a*, and the sum of *bphA1b-nahA1* and *etbA1ab* in strain WAY2, was determined on the mRNA level by RCA.

Separate cultures of the strains C1 and WAY2 were prepared according to a modified protocol from Zubrova *et al.* [38]. Briefly, cells were inoculated in 250 ml Erlenmeyer flasks with 50 ml of LB medium and cultivated O/N. The cell suspensions were harvested by centrifugation, washed and resuspended in 1 l Erlenmeyer flasks containing 250 ml of MSS amended with 30 mM of fumarate to achieve the initial OD_600_ of 0.05. Cultivation proceeded (28 °C, 130 r.p.m.) to the mid-exponential growth phase at which the cells were harvested by centrifugation and washed with a physiological solution. Cells were resuspended in MSS to reach the final OD_600_ of 3 for strain C1 and OD_600_ of 1 for strain WAY2. The bacterial suspensions (10 ml) were distributed into 100 ml Erlenmeyer flasks and amended with 0.5 mM of a single SPM or biphenyl. Prior to the cell addition, the ethanol from the SPM stock solutions was evaporated except for monoterpenes, which were added directly due to their high volatility and liquid state. Biphenyl was employed as a model inducer, whereas an unamended flask served as the reference with no effector, i.e. control. After incubation (6 h for the strain C1 and 3 h for the strain WAY2 according to pilot experiments), 2 ml of the cell suspensions was harvested by centrifugation (13 000 relative centrifugal force (RCF), 10 min and 4 °C) and stored at −80 °C prior to further analysis.

Total RNA isolation was performed by Monarch® Total RNA Miniprep Kit (New England Biolabs, USA) with the following modifications. Enzymatic lysis of pelleted cells was performed by 350 µl of Tris-EDTA buffer (RNase-free grade, Sigma-Aldrich, USA) amended with 3 mg ml^−1^ of lysozyme (Promega, USA), and 15 µl of proteinase K (Zymo Research, USA), and incubated for 15 min on a bench vortex. Mechanical disruption followed in 2 ml tubes (SARSTEDT, GE) with acid-washed glass beads (⁓ 0.5 g, RNase-free grade, Merck, USA), and 200 µl of DNA/RNA Protection Reagent (2×) using MPBIO FASTPREP 24 homogenizer (MP Biomedical, UK), with the use of program 1 (S 6.5; TH BG; T 60) for 5 min. Then, the cell debris was separated by centrifugation (2 min, 16 000 RCF), and 350 µl of the supernatant was transferred into a fresh epi-tube with 350 µl of RNA lysis buffer and vortexed. RNA binding, washing and elution were performed according to the manufacturer’s protocol. Residual DNA was digested by DNase I, and the completeness of DNA removal was verified by quantitative PCR (qPCR) targeting 16S rRNA. The RNA was further concentrated and purified by RNA Clean and Concentrator Kit protocol (Zymo Research, USA) following the manufacturer’s protocol and was eluted into 15 µl of molecular biology-grade water. The quality and quantity of RNA samples were determined by Agilent RNA 6000 Nano Kit (Agilent Technologies, USA) in combination with spectrophotometer NanoPhotometer P330 (Implen, DE). Only the samples with RNA integrity number >7 were employed further for reverse transcription (RT) reaction. RT was performed by 200 U of M-MuLV RT (New England Biolabs, USA) according to the manufacturer’s recommendations with 150 ng of RNA in the total volume of 20 µl of molecular biology-grade water. The resulting cDNA samples were 10× diluted and aliquoted for the assumed number of qPCRs. Samples intended for 16S rRNA gene amplification were diluted 100×.

The qPCR analysis was conducted using a CFX Connect Real-Time PCR Detection System (Bio-Rad, USA), employing the KAPA SYBR FAST qPCR Master Mix (2×) Kit (KAPA Biosystems, USA). A whole reaction volume of 12 µl included specific primers (with concentration stated in Table 1) and 1 µl of diluted cDNA as a template (see above). Primers targeting two variants of the putative naphthalene dioxygenase (NDO), i.e. *nahA1* and *bphA1b* genes, together with two variants of the putative ethylbenzene dioxygenase (EBDO), i.e. *etbA1a* and *etbA1b* genes in strain WAY2, were designed to target both variants of these genes due to their high sequence similarity. DNA of the selected strain previously isolated and cleaned with PureLink™ Genomic DNA Mini Kit (Invitrogen, USA) and Genomic DNA Clean and Concentrator™ Kit (Zymo Research, USA), respectively, was used for the calibration curve. The conditions of the qPCR programme were as follows: (1) 95 °C, 3 min; (2) 95°, 20 s; (3) Table 1, 20 s; (4) 72 °C, 10 s; (5) 72 °C, 2 min. For the amplification, 32–36 cycles were used. Each sample was prepared in three biological replicates each consisting of technical triplicates. To assess the batch effect, each qPCR run included a single biological replicate of all samples and controls.

**Table 1.** Primers used in this study

Primer	Sequence	Strain	Annealing temp. (°C)	concn (µm)
*16S* F	5′-GTAAACGGTGGGCGCTAGGTGTG-3′	WAY2	63	0.40
16S R	5′-CGAATTAATCCACATGCTCCGCC-3′			
*16S* F	5′-GGATTAGATACCCTGGTAGTCC-3′	C1	56	0.40
16S R	5′-CCCCGTCAATTCCTTTGAG-3′			
*bphA1* F	5′-ATCCAGTACCGCGCAACC-3′	C1	60	0.25
*bphA1* R	5′-CGGATGGTGTTGATGCCTGG-3′			
*bphA1a* F	5′-GGCGCTGATTGATCTCCG-3′	WAY2	61	0.25
*bphA1a* R	5′-CGTTGATCCATCAGTGGTGC-3′			
*bphA1b*-*nahA1* F	5′-CCCGCTGGACTACGCCG-3′	WAY1	61	0.25
*bphA1b*-*nahA1* R	5′-CGCCTCCGCTTTCGCTGG-3′			
*etbA1ab* F	5′-GGAGCATGTTGAGGTCTGAGCG-3′	WAY2	61	0.25
*etbA1ab* R	5′-CACCCGAATGGAATTATCCCTG-3′			
*gyrB* F	5′-GTCGAGATCACCCTTCTACCGAG-3′	C1	60	0.25
*gyrB* R	5′-CGGTGTAGGTCTGGTTCCATT-3′			
*gyrB* F	5′-CGGAGTTCCCACTGTCGAGGTG-3′	WAY2	63	0.25
*gyrB* R	5′-GCTCGCCGGGCTTGGAATCC-3′			
*nahA* F	5′-CGCAGTATCACCCGTGTTC-3′	C1	56	0.25
*nahA* R	5′-GCATCCAGTATTCGAGGAAGT-3′			
*phtA1* F	5′-GGATGGAGCAACCTACTGGG-3′	C1	58	0.25
*phtA1* R	5′-GTTGTGGACGAAGCTCAGG-3′			

To quantify the level of gene expression, data were processed via the common-based method [39] using the levels of 16S RNA gene and *gyrB* expression for data normalization. Samples prepared with no effector were analysed as the reference, i.e. control. Two-way ANOVA was performed to test for effector and batch effects. To determine which effector was significantly induced compared to a control and to calculate corresponding 95% CIs, the Dunnett post-hoc test from the R package multicomp was performed. Upon observing high but non-significant induction with a large CI of *p-*cymene with strain C1 and (*R*)-carvone with strain WAY2, another four replicates (seven replicates in total) including biphenyl and controls were measured and tested by t-test. All tests were performed with *α*=0.05.

## Results

### Phylogeny of ARHDs in strains C1 and WAY2

**Fig. 2.**
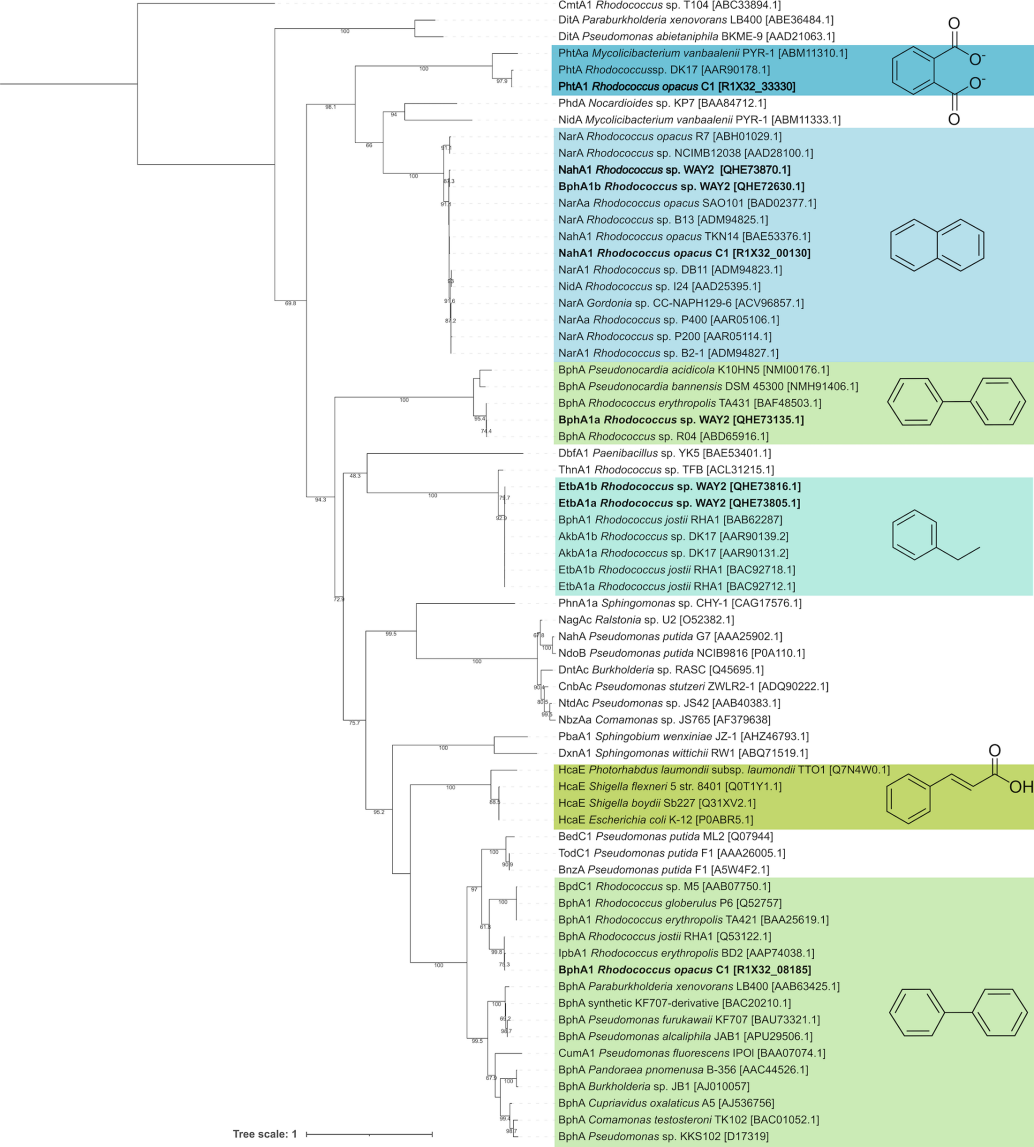
Phylogeny reconstruction of the LSUs of terminal dioxygenases of ARHDs including BphA1, NahA1 and PhtA1 in *R. opacus* C1 and BphA1a, NahA1, BphA1b and EtbA1ab of *Rhodococcus* sp. WAY2. Only bootstrap values >40 are indicated.

**Fig. 3.**
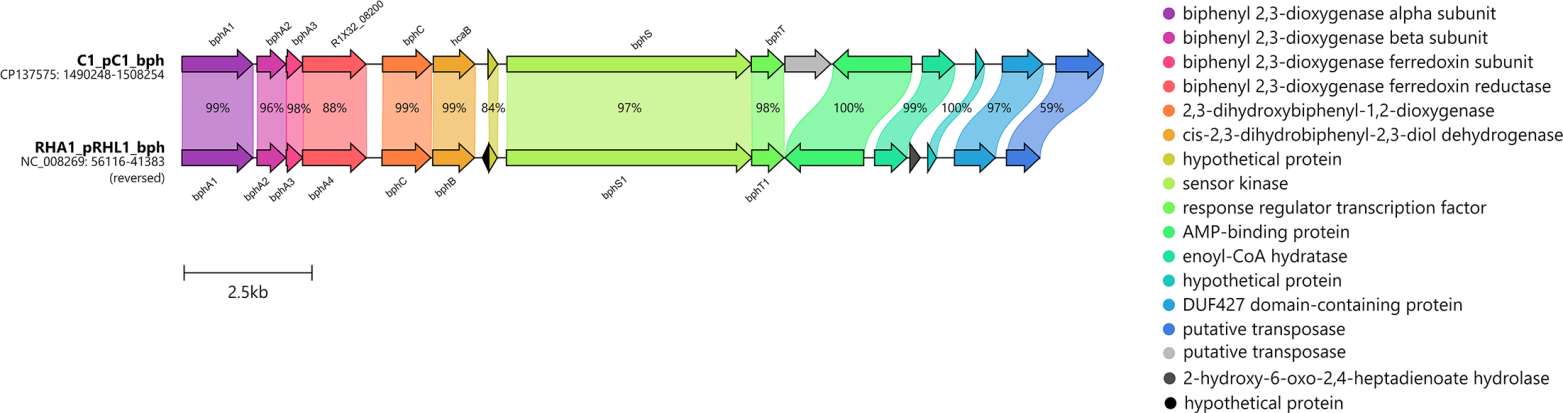
Gene organization of *bph* gene cluster in *R. opacus* C1 and the comparison with homologous *bph* cluster in *Rhodococcus jostii* RHA1. Putative transposases are located upstream and downstream of the C1 strain’s *bph* gene cluster. Percentages indicate the aa sequence identity.

Genomic analysis (Fig. S1) of strain C1 revealed the presence of one linear chromosome (8 002 894 bp) and three linear plasmids (1 727 788 bp, 536 566 bp and 244 600 bp) with an average GC content of 67%. The analysis also revealed a large number of putative ARHD genes (Table S1), including biphenyl dioxygenase (BPDO), NDO and phthalate dioxygenase (PDO), whose LSUs of terminal dioxygenases containing a catalytical domain are encoded by *bphA1*, *nahA1* and *phtA1*, respectively. In the strain WAY2, Garrido-Sanz *et al.* [29] presented a similar set of five ARHD genes, including *bphA1a*, *bphA1b*, *nahA1*, *etbA1a* and *etbA1b*, the latter two of which encode EBDOs. Phylogenetic reconstruction of all these large ARHD subunits shows that they belong to five distinct orthologue groups (Fig. 2). The architecture of the gene clusters containing ARHD-encoding sequences in strains C1 and WAY2 was compared with that of their closely related orthologues from bacteria with available genomes or DNA sequences obtained from GenBank (Figs3^5^).

**Fig. 4.**
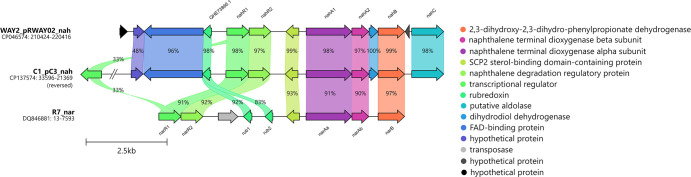
Gene organization of *nah* gene cluster in *R. opacus* C1 together with *nah* cluster in *Rhodococcus* sp. WAY2 and the comparison with homologous *nah* cluster in *R. opacus* R7. Percentages indicate the aa sequence identity.

**Fig. 5.**
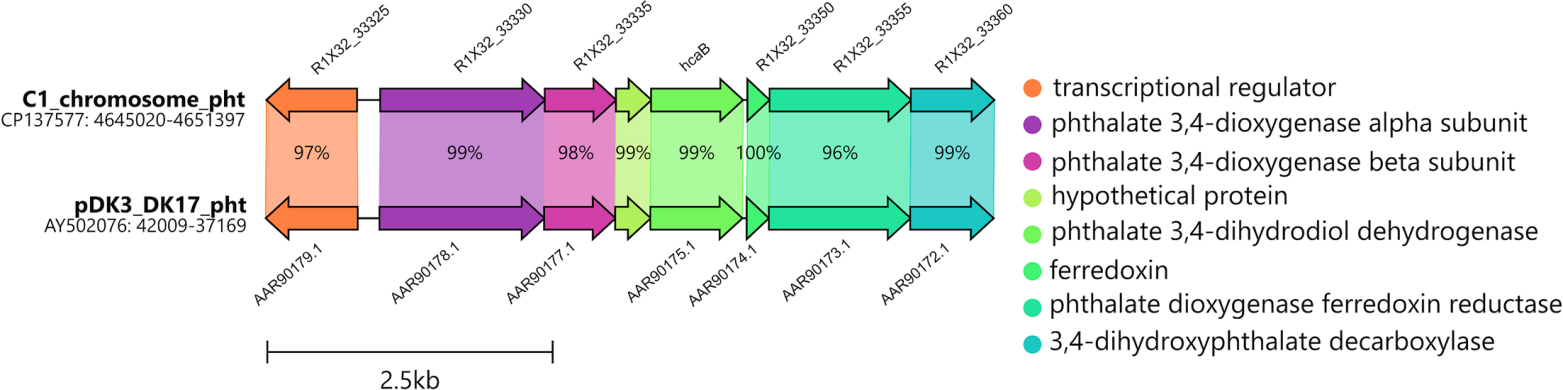
Gene organization of *pht* gene cluster in *R. opacus* C1 and the comparison with homologous *pht* cluster in *Rhodococcus* sp. DK17. Percentages indicate the aa sequence identity.

BphA1 of strain C1, which is encoded on plasmid pC1, clusters with those of *R. erythropolis* BD2 [40] and the model PCB degrader *R. jostii* RHA1 [41] (Fig. 2). In fact, the entire *bph* region architecture in strain C1 is highly similar to that of RHA1, and their aa sequences share 99% identity (Fig. 3). NahA1 of the strain C1, encoded on plasmid pC3, clusters with other, mostly rhodococcal NDO LSUs (Fig. 2). Within this phylogenetic clade, the two putative NDO LSU variants NahA1 and BphA1b of strain WAY2 are also found (Fig. 2) (sharing 99.4% of identity, for more details, see [21]), both of which share 98.5 and 98.7% identity, respectively, with NahA1 of strain C1 (Fig. 4). Furthermore, the architecture of the *nah* regions in C1 and WAY2 is mostly identical (Fig. 4). PhtA1 of strain C1, which is encoded on the chromosome, clusters with PhtA1 of *R. opacus* DK17 [42] and PhtAa of *Mycolicibacterium vanbaalenii* PYR-1 [43], classified as PDO (Fig. 2). PhtA1 of strains C1 and DK17 share 99% sequence identity; in fact, the architecture of both gene regions is highly similar (Fig. 5).

The remaining ARHDs in strain WAY2 were also included in the phylogeny reconstruction (Fig. 2), i.e. BphA1a, EtbA1a and EtbA1b (for more details, see [21]). BphA1a of WAY2 together with BphA1 of *R. erythropolis* TA431 and *Rhodococcus* sp. R04 [44] and BphA1 of strain C1 form a separate cluster distant from the most studied BPDOs (Fig. 2). The two putative EBDO LSU variants EtbA1a and EtbA1b of WAY2, whose genes typically differ by one base and share 99.8% aa identity, cluster with EtbA1a and EtbA1b of strains RHA1 [41] and AkbA1a and AkbA1b of *Rhodococcus* sp. DK17 (Fig. 2), which encode for LSU of alkylbenzene dioxygenases [45].

### Utilization and transformation of SPMs and biphenyl by the strains C1 and WAY2

Both strains C1 and WAY2 were tested for the utilization of selected SPMs and biphenyl as a sole carbon source as well as the transformation of these substrates. The strains’ ability to transform SPMs was determined by analysing the compound depletion by viable or autoclaved cells using GC-MS or LC-UV/VIS analyses.

Whilst both strains were able to transform biphenyl, only strain WAY2 was able to utilize it as the sole carbon source (Table 2). On the other hand, strain WAY2 was not able to use any of the tested SPMs as the sole carbon source, whilst it transformed flavone, flavanone and monoterpenes (Table 2). Strain C1 was able to utilize four of the tested flavonoids as well as a monoterpene *p*-cymene. Furthermore, two other monoterpenes were transformed by strain C1 (Table 2). The additional information on the ability of strain C1 to utilize a wider range of SPMs and naphthalene is provided in Table S2. Similar information for strain WAY2 is provided by Garrido-Sanz *et al.* [21].

**Table 2.** Utilization (U) and transformation (T) of SPMs and biphenyl by strains C1 and WAY2. When no utilization was observed, the strains’ ability to transform the compound was explored further. SPM transformation was tested on the basis of the compound depletion by viable and autoclaved cells (t-test, *α*=0.05)

	Substrate	Strain C1	Strain WAY2
	Biphenyl	**T**	**U**
Flavones	Flavone	–	**T**
	Luteolin	–	–
Flavanones	Flavanone	**U**	**T**
	Naringenin	**U**	–
Flavonols	Kaempferol	–	–
	Quercetin	–	–
Flavan-3-ol	Catechin	**U**	–
Isoflavone	Daidzein	**U**	–
	Genistein	–	–
Monoterpenes	(*S*)*-*Limonene	**T**	**T**
	*α*-Pinene	**T**	**T**
	(*R*)-Carvone	–	**T**
	*p-*Cymene	**U**	**T**

### Transcription of ARHD genes in strains C1 and WAY2 in the presence of SPMs and biphenyl

**Fig. 6.**
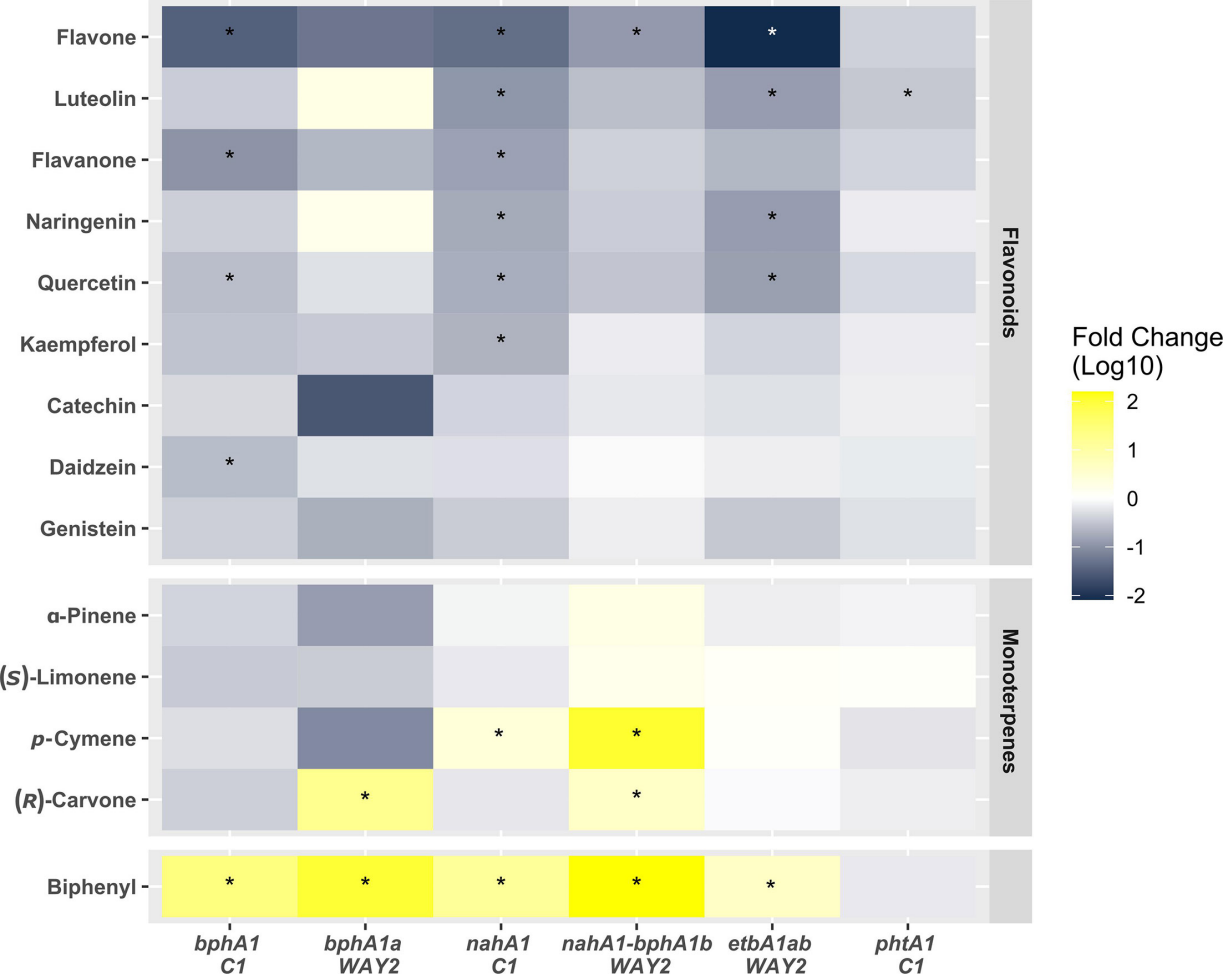
The relative level of transcripts of ARHD genes in strains C1 (*bphA1*, *nahA1* and *phtA1*) and WAY2 (*bphA1a*, *nahA1-bphA1b* and *etbA1ab*) after the exposure to biphenyl or SPMs. The relative transcription levels were processed by the common-based method [39], using 16S rRNA and *gyrB* genes as reference genes. Data were analysed by ANOVA with the Dunnett post-hoc test. The relative levels of ARHD transcripts in strain C1 after the exposure to *p-*cymene and in strain WAY2 after the exposure to (***R***)*-*carvone and biphenyl were tested by t-test. Data are presented in log_10_ scale. An asterisk indicates a significant difference in the relative level of gene transcripts compared to the unaffected cells, i.e. control (*α*=0.05).

To reveal whether SPMs affect the transcription of the ARHD genes in strains C1 and WAY2, the relative level of ARHD gene transcripts was quantified upon exposure to SPMs as summarized in Figs 6 and S2–S5.

In strain C1, the *bphA1* gene transcription was induced upon exposure to biphenyl, with a 24-fold increase in transcripts compared to the unaffected control. SPMs mainly inhibited *bphA1* gene transcription, with flavone showing a 30-fold decrease compared to the control, as well as for flavanone (tenfold), quercetin (fourfold) and daidzein (fourfold). The *nahA1* gene transcription in strain C1 was induced by biphenyl (14-fold) and by *p-*cymene (10-fold); its inhibition was observed for flavone, with a 23-fold decrease in transcripts compared to the control, as well as for flavanone, luteolin, naringenin, kaempferol and quercetin, ranging from a five- to ninefold decrease. The *phtA1* gene transcription in strain C1 was inhibited by luteolin, showing a threefold decrease compared to the control. The remaining compounds left *phtA1* transcription unaffected (Figs 6, S2 and S3).

In strain WAY2, *bphAa* gene transcription was induced upon exposure to biphenyl, with a 69-fold increase compared to the unaffected control, and a 12-fold increase after exposure to (*R*)*-*carvone. The sum of *nahA1-bphA1b* transcripts in strain WAY2 was increased by biphenyl, showing a 188-fold increase compared to the control, as well as for *p-*cymene (111-fold) and (*R*)*-*carvone (3-fold); the decrease of *nahA1-bphA1b* transcripts was observed upon exposure to flavone, showing an eightfold decrease compared to the control. The sum of *etbAab* transcripts in strain WAY2 was increased by biphenyl (fourfold), whereas it was decreased by flavone, showing a 122-fold decrease compared to the control, as well as for luteolin, naringenin and quercetin, showing around a sevenfold decrease compared to the control (Figs 6, S4 and S5).

## Discussion

ARHDs play a crucial role in the aerobic biodegradation of both natural and anthropogenic aromatic compounds [33,38, 46,49]. Due to the multiple copies and variants of ARHDs carried in their extensive genomes, rhodococci can thrive in diverse environments, ranging from plant rhizospheres to polluted sites, using a variety of aromatic compounds as sources of carbon and/or energy [23,25,50]. The ability of ARHDs to process aromatic pollutants has supposedly evolved from the transformation of structurally similar SPMs [4,8, 51, 52], possibly by exaptation of the respective enzymes [33]. Thus, we investigated the capacity of two *Rhodococcus* strains, C1 and WAY2, which both originate from soil contaminated with aromatic pollutants, to utilize SPMs and explored the role of SPMs in the expression of selected ARHDs found in their genomes. Whilst strain WAY2 originates from soil contaminated with polycyclic aromatic hydrocarbons (PAHs) [29], strain C1 was isolated from a chlorophenol-, chlorinated dibenzofuran- and dibenzodioxin-contaminated site. Furthermore, strain C1’s habitat was heavily enriched with lignocellulose, which might imply the ability of strain C1 to utilize some of the tested SPMs, i.e. flavanone, naringenin, catechin, daidzein and *p-*cymene (Table 2) and other phenolic compounds stated in Table S2. Strain WAY2 did not utilize any of the tested SPMs but transformed all of the tested monoterpenes and non-hydroxylated flavonoids, including flavone and flavanone (Table 2). Whilst some rhodococcal strains have been reported to transform or even utilize monoterpenes such as limonene, carvone or *p*-cymene [51,53,56], to date, only *R. erythropolis* U23A has been reported to transform flavanone. Furthermore, to the best of our knowledge, none of the *Rhodococcus* strains has yet been reported to utilize flavonoids tested in this study (Fig. 1, Table 2).

The LSUs of several phylogenetically distinct ARHDs from strains C1 and WAY2 were investigated regarding their up/downregulation by SPMs (Figs2  6). The LSU or *α*-subunit is a crucial part of BPDO’s terminal dioxygenase: its substrate-binding domain allows ARHDs to accommodate a remarkably broad range of substrates [33,38, 41, 57]. BPDO, one of the most studied ARHDs, is responsible for initiating PCB degradation. Based on the phylogenetic analysis, BphA1 from strain C1 (gene coordinates R1X32_08185) clusters with other BphA1s from Gram-positive bacteria (*R. erythropolis* BD2, *R. jostii* RHA1) separately from BphA1s from well-known PCB degraders from Gram-negative bacteria, such as *Paraburkholderia xenovorans* LB400, *Metapseudomonas furukawaii* KF707 and *Pseudomonas* sp. KKS102 (Fig. 2) [58,60]. BphA1 from strain RHA1 (GenBank Q53122.1) together with IpbA1 from *R. erythropolis* BD2 (GenBank AAP74038.1) are the closest homologues of BphA1 from strain C1 (Fig. 2). Although there is a high similarity in the architecture of the *bph* region within strains C1 and RHA1 (Fig. 3), the upper biphenyl pathway is incomplete in the genome of the former. Specifically, strain C1 lacks the *bphD* gene encoding for 2-hydroxy-6-oxo-6-phenylhexa-2,4-dienoate hydrolase, which catalyses the degradation of the *meta*-cleavage product 2-hydroxy-6-oxo-6-phenyl-2,4-dienoate (HOPDA). This causes the inability of strain C1 to use biphenyl as a growth substrate (Table 2) and the accumulation of yellow HOPDA in the medium. Such a phenomenon has also been observed in strain BD2 with a missing *ipbD* orthologue [40,61]. The fact that both *bph* and *ipb* gene clusters are surrounded by transposases in both strains, C1 and BD2 [40], respectively, indicates their likely acquisition by horizontal gene transfer. This observation is also supported by the analogous GC content of the *bph* regions of both strains, i.e. 63.35% (located on pBD2 15149–163300) and 63.41% (located on pC1 1490274–15002), in contrast to the genomic GC content of strain C1, i.e. 66.82%.

Even though biphenyl is not utilized by strain C1, it efficiently induces *bphA1* gene transcription (Fig. 6), which has also been reported in strain RHA1 [62]. However, the extensive inhibition of *bphA1* gene transcription by flavonoids, i.e. flavone, flavanone, quercetin and daidzein (Fig. 6), may seem to contradict the following studies: Pham *et al.* [15] reported that *bphA1* gene transcription in *R. erythropolis* U23A was induced by isoflavone. They also reported upregulation of the *bph* pathway due to the higher degradation of 4-chlorobiphenyl in the presence of all tested flavonoids (concentration of 100 µm), some of which were tested in our study, namely flavone, flavanone, naringenin, catechin, kaempferol and quercetin. Using the same approach, Pham *et al.* [63] reported the upregulation of the *bph* pathway in *Pandoraea pnomenusa* B356 by isoflavone, whereas in *Paraburkholderia xenovorans* LB400, the upregulation was not observed for any of the tested flavonoids, i.e. flavone, flavanone and isoflavone. In contrast, Ghitti *et al.* [64] reported the induction of *bphA* gene transcription in strain LB400 by 100 µm flavone and quercetin. In our previous study, we also reported the induction of *bphA* genes in *E. alcaliphila* JAB1 by flavanone, naringenin, quercetin and catechin, whereas inhibition was observed for flavone, as well as for naringenin with a longer incubation period [38]. These seemingly contradictory results may be explained by the divergent phylogeny within the BphA clade, which clearly distinguishes two separate branches of Gram-positive and Gram-negative bacteria (Fig. 2). Diverse evolutionary events within the BphA in Gram-positive bacteria likely gave rise to separate branches, such as in the case of BphA1 from strain C1 and the BphA1 from *Rhodococcus globerulus* P6 (GenBank Q52757) (Fig. 2), or the BphA1 of strain U23A, whose sequence is not available but is reported by the authors to share 99% identity with the BphA1 sequence from strain P6 [16]. Thus, a variable response of BphA expression to the presence of SPMs in rhodococci may have resulted from their divergent evolution/adaptation. Additionally, different experimental designs across the above-mentioned studies prior to RNA isolation such as (i) growing or RCA [15,38, 63,65], (ii) growth substrate [15,65] and (iii) SPM co-incubation time [38,65] should be considered as ulterior factors that may affect the eventual findings. Finally, the regulatory systems of gene expression should be taken into account. For instance, BphST, a two-component regulatory system of a sensor kinase (BphS) and a response regulator (BphT), is involved in the regulation of the *bph* region in Gram-positive bacteria such as *R. jostii* RHA1 (reviewed by Fujihara *et al.* [66]), which was also detected in C1 strain’s genome (Fig. 3). Moreover, a 24-bp-long sequence, which is responsible for the *bphST* gene regulation [67,68], is consensual within the *bph* region of the C1 strain, located 185 bp upstream of the *bphA1* gene. In fact, using heterologous expression, Takeda *et al.* [69] found that *p*-cymene upregulated the expression of BphST in strain RHA1, thereby implying possible induction of the *bphA1* gene. However, the *bphA1* gene induction in strain C1 was not observed in the presence of *p-*cymene. Multiple regulatory systems controlling the expression of the same gene region were reported, and, vice versa, one regulatory system affecting multiple gene regions, such as BphS1T1 in RHA1 affecting both *bph* and *etb* regions [67,70]. Moreover, Takeda *et al.* [67] also observed a duplicity of the BphST regulatory system with a diverse response to biphenyl as an inducer. Therefore, the contradictory response of the transcription of *bphA1* genes to *p-*cymene in the wild-type strain C1, or flavonoids in the abovementioned wild-type strain U23A [15], might also result from a cross-talk among multiple regulatory systems.

The BphA1 of strain WAY2 forms a rather distinct branch in the phylogenetic tree and clusters with BphAs from *Rhodococcus* sp. R04 (GenBank ABD65916.1) and *R. erythropolis* TA431 (GenBank BAF48503.1) (Fig. 2). Strain TA431 was isolated from termite gut along with *R. rhodochrous* K37 and *Rhodococcus* sp. HA99 [50]. The *bph* regions of these isolates are considered atypical, possibly due to the separate evolutionary paths, although they still showed the typical induction of *bphA* gene transcription by biphenyl [71]. Interestingly, strain R04, along with strain WAY2, was reported as a versatile PCB degrader [21,72], whereas strain TA431 was found to have a low affinity to PCBs and was not further investigated [50]. In contrast, *R. erythropolis* TA421, also isolated from termite gut, degrades a wide range of PCBs and harbours BphA1, which belongs to the typical PCB degraders [50] (Fig. 2). Our results revealed that the rather atypical *bphA1* gene of strain WAY2 is not only induced by biphenyl but also by (*R*)-carvone (Fig. 6), a substrate, which was also reported to enhance PCB biodegradation by other bacteria [73,75]. Although insignificant, the remaining monoterpenes and most of the flavonoids appear to have an inhibitory effect on the *bphA1* transcription (Fig. 6).

EBDO is another enzyme involved in the catabolism of biphenyl and PCBs [62]. Whilst the BPDO in strain RHA1 preferentially transforms lower halogenated biphenyls, EBDO transforms the higher halogenated ones [41,76]. In the WAY2 genome, there are two variants of EtbA1a and EtbA1b (QHE73805.1 and QHE73816.1) that differ only in one base. This arrangement is also present in strain RHA1 (BAC92712.1 and BAC92718.1) [77] and *Rhodococcus* sp. DK17 (AAR90131.2 and AAR90139.2) [45], whose both EtbA1s form one branch in the phylogenetic tree (Fig. 2). The transcription of the sum of *etbA1ab* genes in strain WAY2 was induced by 0.5 mM biphenyl, resulting in a 4.4-fold increase in transcripts. Gonçalves *et al.* [62] reported that *etbA1ab* genes in RHA1 were induced ~20 times more, but with proportionally higher biphenyl concentrations. However, when the strain WAY2 was exposed to the same concentration of flavone, the *etbA1ab* genes were inhibited 122-fold. Luteolin, naringenin and quercetin also inhibited the *etbA1ab* genes, although not to such an extent (Fig. 6).

Although NDOs are primarily recognized for the ability to degrade PAHs, they have also been reported to play a role in the catabolism of both PCBs [78,80] and flavonoids [81]. In Gram-negative bacteria, the genes of the *nah* region are typically organized into one or two operon units and are regulated by LysR-type proteins [82,83]. On the other hand, Gram-positive bacteria, such as rhodococci, usually harbour three to four structural genes (*narA1A2B/narA1A2BC*) distributed across several homologous transcriptional units and are regulated by GntR-type proteins [21,84, 85]. In the strain WAY2, the *nahA1A2BC* region is located on a plasmid pRWAY02, and its homologous *bphA1bA2bBC* region is located on plasmid pRWAY01 [21]. The architecture of these regions is highly similar to the *nah* region of strain C1 (Fig. 4). Finally, since NahA1/BphA1b in strains C1 and WAY2 share a high percentage of identity (Fig. 4), their transcriptional profiles reflect the inhibitory effect of most of the tested flavonoids, whilst the induction is observed for both biphenyl and *p-*cymene (Fig. 6). Moreover, (*R*)-carvone induced the transcription of the sum of *nahA1-bphA1b* genes in strain WAY2.

PhtA1 is the LSU of PDO, which is another protein in the diverse family of ARHDs. PDOs incorporate the oxygen atom into the aromatic structure of various compounds, including phthalate, vanillate, chlorobenzoate, phenoxybenzoate and *p*-toluenesulfonate [86]. Interestingly, whilst PDOs in Gram-negative bacteria are mostly known to have only the LSU, i.e. *α*-type [43,87, 88], PDOs in Gram-positive bacteria comprise both the large and small subunits, i.e. *αβ*-type [42,89], which are present in all other ARHDs discussed herein (Figs3^5^). Furthermore, the PDOs of *Comamonas testosteroni* KF1 and *Burkholderia cepacia* DB01 catalyse the dihydroxylation of phthalate to 4,5-dihydrodiolphthalate [88], whereas the PDOs of RHA1 and DK17 hydroxylate phthalate to 3,4-dihydrodiolphthalate [42,89]. Our results show that the expression of PhtA1 in strain C1, which clusters with OphA1 of strain DK17 (Fig. 2) with identical *pht*/*oph* region architecture (Fig. 5), was not affected by most of the compounds tested, including biphenyl. Only luteolin slightly downregulated *phtA1* expression (Fig. 6). To the best of our knowledge, our study is the first to report the effect of flavonoids and monoterpenes on the expression of NahA1/NDO, EtbA1/EBDO or PhtA1/PDO.

In summary, our results add another missing piece to the puzzle of how SPMs relate to ARHDs. Flavonoids and monoterpenes have been demonstrated to be inducers of biodegradative pathways in soil consortia, resulting in higher degradation of PCBs and PAHs [4,10, 16, 51, 73, 75, 90]. Our data, along with the results of multiple previous studies [15,33, 38, 49, 63, 64, 69], provide strong support for these conclusions at the molecular level. Nevertheless, the enormous variability of both ARHDs and SPMs calls for further research, similar to that presented herein, to gain a more comprehensive understanding of the relationship between these molecules. Initially, ARHDs were grouped into four main categories that catabolize toluene/biphenyl, naphthalene, benzoate and phthalate [86]. Subsequently, Iwai *et al.* [2] extended the classification of PAH dioxygenases into Gram-positive and Gram-negative clades. In terms of primer design, Meynet *et al.* [91] specifically focused on the *α*-subunit of ARHDs and distinguished 20 groups. More recently, Garrido-Sanz *et al.* [92] analysed the diversity of ARHDs only within the genus *Rhodococcus* and identified 339 non-identical ARHDs, of which the substrate specificity of more than 200 ARHDs remains unknown. Despite this, the current results suggest that some ARHDs may have evolved from enzymes that originally had degraded SPMs. Alternatively, some of the ARHDs in rhodococci may have diverged earlier and evolved to catabolize pollutants in their environment, which may have posed a greater threat than antimicrobial SPMs. Consequently, these ARHDs have a lower affinity to the SPMs. Our results highlight the complexity of microbial adaptation to environmental challenges with insights into the molecular basis and call for further research in this area. It is also important to consider broadening the research approach to include a wider range of enzymes and substrates, beyond those historically focused on the metabolism of pollutants, as in this study.

## supplementary material

10.1099/mgen.0.001359 Uncited Supplementary Material 1.

## Abbreviations

ARHDs: aromatic ring-hydroxylating dioxygenases
BPDO: biphenyl dioxygenase
EBDOs: ethylbenzene dioxygenase
HOPDA: 2-hydroxy-6-oxo-6-phenyl-2,4-dienoate
LB: Luria Broth
MSS: minimal salt solution
NDO: naphthalene dioxygenase
PAHs: polycyclic aromatic hydrocarbons
PCA: plate count agar
PCBs: polychlorinated biphenyls
PDO: phthalate dioxygenase
qPCR: quantitative PCR
RCA: resting-cell assay
RCF: relative centrifugal force
RT: reverse transcription
SPMs: secondary plant metabolites
