# Supplementary material for: Differential effect of monoterpenes and flavonoids on the transcription of aromatic ring-hydroxylating dioxygenase genes in Rhodococcus opacus C1 and Rhodococcus sp. WAY2
Source: Microb Genom. 2025 Mar 5;11(3):001359. doi: 10.1099/mgen.0.001359 (PMC11881993; doi:10.1099/mgen.0.001359)
Supplement: Uncited Supplementary Material 1. [file mgen-11-01359-s001.pdf]

Differential Effect of Monoterpenes and Flavonoids on the Transcription of Aromatic Ring-Hydroxylating Dioxygenase Genes in *Rhodococcus opacus* C1 and *Rhodococcus* sp. WAY2

Andrea Zubrova[1*], Manuela Tadrosova[1*], Jaroslav Semerad[2,3], Tomas Cajthaml[2,3], Petr Pajer[4], Michal Strejcek[1], Jachym Suman[1], Ondrej Uhlik[1✉]

[1]*University of Chemistry and Technology, Prague, Faculty of Food and Biochemical Technology, Department of Biochemistry and Microbiology, Prague, Czech Republic*

[2]*Institute of Microbiology, Academy of Sciences of the Czech Republic, v.v.i., Prague, Czech Republic*

[3]*Charles University, Faculty of Science, Institute for Environmental Studies, Prague, Czech Republic*

[4]*Military Health Institute, Ministry of Defence of the Czech Republic, U Vojenske nemocnice 1200, 169 02 Prague 6, Czech Republic*

✉**Corresponding authors:** University of Chemistry and Technology, Prague, Technicka 3, 166 28 Prague 6, Czech Republic. Phone: +420 220 44 2020; email: ondrej.uhlik@vscht.cz

Supplementary material

Genome analysis of strain C1

The culture was grown overnight on PCA at 28°C. Genomic DNA was isolated with a FastDNA SPIN Kit (MPBio, USA) following the manufacturer's protocol. Sequencing libraries were prepared using rapid sequencing kit (Oxford Nanopore) and sequenced on a Nanopore MinION paltform (FLO-MIN106). In total, 578 328 Nanopore reads (average read length, 4 729.5 bp; the total number of bases, 2.7 Gbp) were generated, corresponding approximately to a 47× genome coverage. The sequencing adaptors were removed using porechop (v.0.2.4) and the reads were further filtered out using Filtlong (v.0.2.1) with options --min_length 1000 --keep_percent 90. The cleaned reads were assembled using Flye (v.2.9.2) [1] with options --nano-corr. The obtained assembly was polished with Medaka (v.1.11.3) with option -m r941_min_hac_g507. The data were submitted to NCBI GenBank under the Bioproject PRJNA1028948.

Genome characteristics

The genome of *Rhodococcus opacus* C1 was obtained with 94.71% completeness and 3.55% redundancy as measured by CheckM (v.1.2.3) [3]. The genome consists of one linear chromosome (8,002,894 bp) with three linear plasmids (1,727,788 bp, 536,566 bp, and 244,600 bp) with an average GC-content of 67% (Fig. S1). The PGAP annotation [4] identified 722 pseudogenes of which 462 were frameshifted, 382 were incomplete, and 49 had internal stop codons. A total of 9,843 genes were identified, including 51 tRNAs and 12 rRNAs (4 5S, 4 16S and 4 23S). Genome maps were generated using PGAP annotation in the GenoVi tool [2]. Taxonomy placement was performed using GTDBtk (v.2.3.0) [5], which identified the closest genome as that of *Rhodococcus opacus* 1b^{T} with 99.16% ANI.

Supplementary Figure S1. Genomic map of *Rhodococcus opacus* C1 replicons [2]. The linear chromosome (CP137577, 67.5 % GC content) and three linear plasmids pC1_1 (CP137575, 65.5 % GC content), pC1_2 (CP137576, 64.5 % GC content), and pC1_3 (CP137574, 65 % GC content) are displayed. The outer two rings represent coding sequences in the forward strands (CDS+, second ring) colored according to the COG (clusters of orthologous groups, first ring) types. Other genomic features, such as tRNA and rRNA, are displayed in the rings below. The inner two rings represent a deviation in the G+C content and G+C skew.

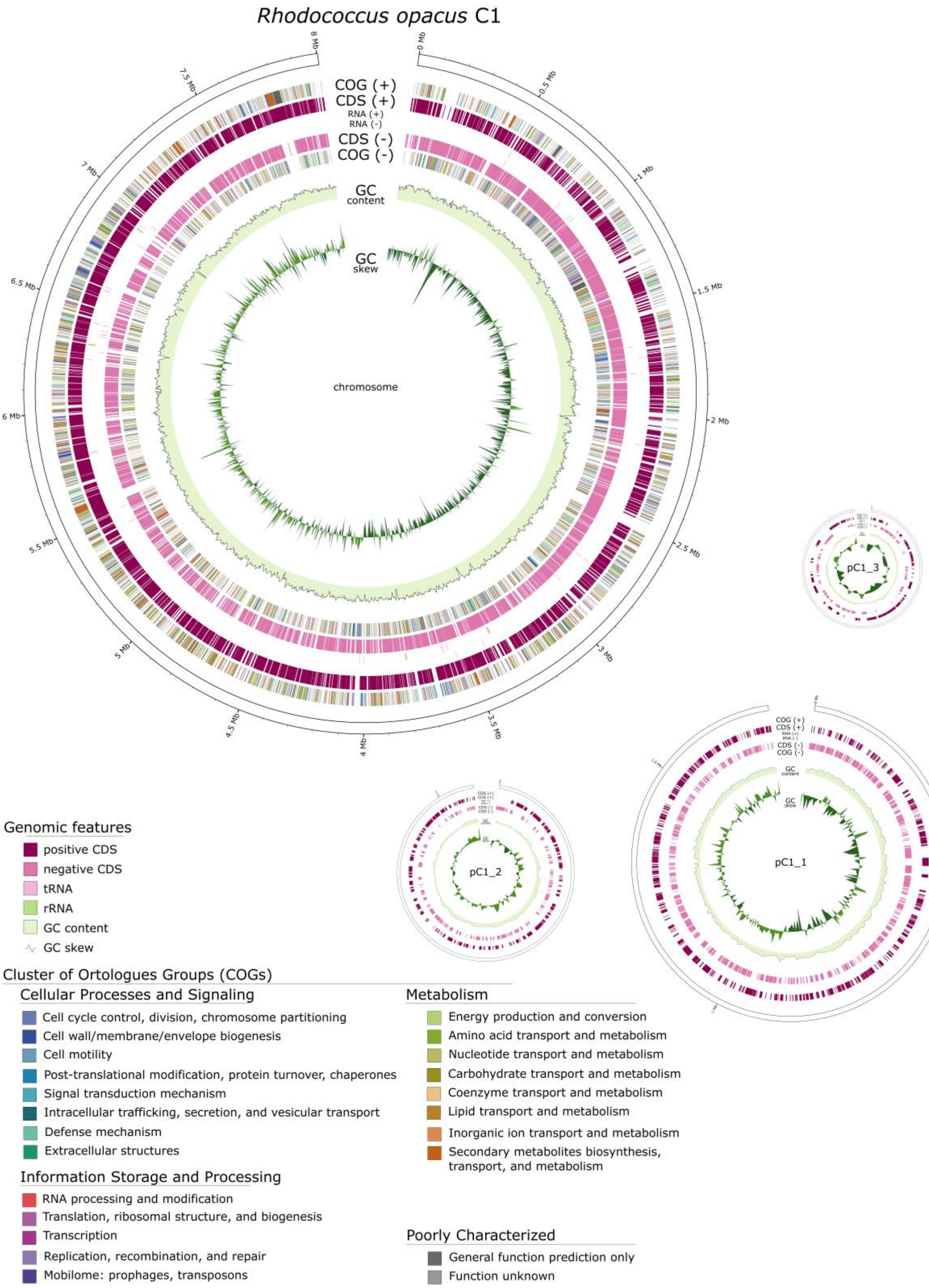
Rhodococcus opacus C1
0 Mb
0.5 Mb
1 Mb
1.5 Mb
2 Mb
2.5 Mb
3 Mb
3.5 Mb
4 Mb
4.5 Mb
5 Mb
5.5 Mb
6 Mb
6.5 Mb
7 Mb
7.5 Mb
8 Mb
COG (+)
CDS (+)
RNA (+)
RNA (-)
CDS (-)
COG (-)
GC content
GC skew
chromosome
pC1_3
pC1_2
pC1_1
Genomic features
positive CDS
negative CDS
tRNA
rRNA
GC content
GC skew
Cluster of Ortologues Groups (COGs)
Cellular Processes and Signaling
Cell cycle control, division, chromosome partitioning
Cell wall/membrane/envelope biogenesis
Cell motility
Post-translational modification, protein turnover, chaperones
Signal transduction mechanism
Intracellular trafficking, secretion, and vesicular transport
Defense mechanism
Extracellular structures
Information Storage and Processing
RNA processing and modification
Translation, ribosomal structure, and biogenesis
Transcription
Replication, recombination, and repair
Mobilome: prophages, transposons
Metabolism
Energy production and conversion
Amino acid transport and metabolism
Nucleotide transport and metabolism
Carbohydrate transport and metabolism
Coenzyme transport and metabolism
Lipid transport and metabolism
Inorganic ion transport and metabolism
Secondary metabolites biosynthesis, transport, and metabolism
Poorly Characterized
General function prediction only
Function unknown

Search for ARHDs

To obtain a complete set of ARHDs in *Rhodococcus opacus* C1 (Table S1), both the ARHD α-subunit and Rieske component HMM profiles (available at UniProt: PF00848.23, PF00355.30 respectively) were simultaneously searched against the C1 genome with HMMER (v.3.4) [6]. Putative functions of the ARHD sequences were then searched using BLASTP against the nr database.

Table S1. Set of ARHDs in *Rhodococcus opacus* C1. ARHD α-subunit and Rieske component HMM profiles were searched against the C1 genome with HMMER. Putative protein specificity was searched by BLAST against nr database.

**Gene**<br>Replicon: gene ID<br>(gene coordinates)	**Identity [%]**	**Accession Number**	**Putative Protein Specificity**	**Organism**	**Citation**
nahA1<br>CP137574:<br>R1X32_00130<br>(23,220-24,626)	98.93	WP_159927768.1	Naphthalene dioxygenase large subunit	*Rhodococcus* sp. WAY2	[7]
bphA1<br>CP137575:<br>R1X32_08185<br>(1,490,248-1,491,630)	99.35	WP_011599002.1	Biphenyl 2,3-dioxygenase subunit alpha	*Rhodococcus jostii* RHA1	[8]
CP137575:<br>R1X32_01475<br>(78,486-79,832)	100	WKN61298.1	ARHD	*Rhodococcus opacus 3*D	[9]
cmtA1<br>CP137575:<br>R1X32_05705<br>(977,693-979,006)	97.71	TQC41472.1	*p*-cumate dioxygenase	*Rhodococcus* sp. WS4	x
CP137576:<br>R1X32_11090<br>(366,323-367,660)	99.2	WP_317571927.1	x	x	x
CP137576:<br>R1X32_11620<br>(501,838-503,016)	55.95	HWI71024.1	ARHD	*Beakduia* sp.	[10]
phtA1<br>CP137577:<br>R1X32_33330<br>(4646,017-4647,462)	98.75	AAR90178.1	phthalate 3,4-dioxygenase subunit alpha	*Rhodococcus* sp. DK17	[11]
tphA1<br>CP137577:<br>R1X32_33285<br>(4,637,988-4,639,253)	99.76	ELB91386.1	terephthalate 1,2-dioxygenase alpha subunit	*Rhodococcus wratislaviensis* IFP 2016	[12]
CP137577:<br>R1X32_34835<br>(4,977,473-4,978,762)	100	WP_005574578.1	x	x	x
benA1<br>CP137577:<br>R1X32_36710<br>(5,386,709-5,388,085)	100	WP_120660447.1	benzoate 1,2-dioxygenase large subunit	x	x
CP137577:<br>R1X32_39665<br>(6,006,694-6,007,866)	100	WP_007300410.1	x	x	x

Utilization of naphthalene and other SPMs by strain C1

Strain C1 was tested for its ability to use a wider range of SPMs and naphthalene as the sole carbon source at a concentration of 1 mM (Tab. S2). Samples were prepared as described in the Materials and Methods section.

Strain C1 was able to utilize naphthalene and most of the tested phenolic compounds, except for 4-hydroxycoumaric acid, salicylic acid, and vanillin (Tab. S2).

Table S2. SPMs and naphthalene utilization by strain C1.

**Group of SPMs**	**Substrate**	**Strain C1**
	Naphthalene	U
Flavonoids	Morin	-
	Chrysin	-
Coumarins	Umbelliferon	-
Phenolic acids	Caffeic acid	U
	Cinnamic acid	U
	Ferulic acid	U
	Gallic acid	U
	p-hydroxybenzoic acid	U
	4-hydroxycoumarin	-
	p-coumaric acid	U
	Protocatechuic acid	U
	Salicylic acid	-
	Vanillin	-

Transformation of SPMs and biphenyl by strains C1 and WAY2

Analysis of flavonoids

High-performance liquid chromatography (HPLC) coupled with a UV-VIS diode array detector was used to analyze flavonoids: flavone, luteolin, flavanone, naringenin, kaempferol, quercetin, catechin, daidzein, and genistein. The analysis was conducted using the NexeraXR system equipped with the SPD-M20A detector (Shimadzu). Prior to the analysis, an equal volume of methanol was added to the glass vials, cell suspensions were sonicated for 15 min, and cell debris was removed by centrifugation. The content of individual flavonoids in the samples was then determined with the HPLC, using the following program: 0-1.2 min – 20% A, 1.5-4 min – gradient of 20%-80% A, 3.5-5.5 min – 95% A, 4-7 min – 80%, 7-8 min gradient of 80% – 20% A, 80-10 min – 20% A; mobile phase A: methanol with 0.02% (v/v) trifluoracetic acid, B: H2O with 0.02% (v/v) trifluoracetic acid; mobile phase flow 0.8 ml/min. A Luna® Omega Polar C18 column (Phenomenex) was used for separation at 40 °C. The detection wavelengths [nm] and corresponding elution times [min] for each compound were the following: flavone 296/7.13, luteolin 366/6.18, flavanone 249/7.00, naringenin 289/6.08 kaempferol 262/6.31, quercetin 370/6.01, catechin 220/2.64, daidzein 248/5.94, genistein 259/6.19. The linearity of the detector response was verified for the concentration range of 1 – 500 µM for all analytes.

Analysis of monoterpenes

Gas chromatography coupled with mass detection (GC-MS) was employed to analyze monoterpenes, including (*S*)-limonene, (*R*)-carvone, *p*-cymene, and *α*-pinene. The analysis was conducted using a Varian 450-GC instrument (USA) with a Combi-Pal autosampler (CTC Analytics, Sweden) and a Varian 240 MS ion trap mass spectrometer. Prior to the analysis, samples were extracted with ethyl acetate (5 ml; 3 times) employing a reciprocating shaker. The extracts were dried with sodium sulfate, concentrated to 1 ml using a nitrogen stream, and analyzed by GC-MS with a sample injection volume of 1 µl. Separation was carried out on a DB-5MS column (Agilent, Germany), which was 30 mm long x 0.25 mm I.D. with a 0.25 µm film thickness. The GC oven temperature program started at 45 °C (held for 6 min), then gradually increased to 80 °C (2 °C/min, held for 5 min), and finally heated up to 200 °C (10 °C/min), where it was held isothermally for 2 min. The GC oven temperature program for the analysis began at 60°C (held for 1 minute) and gradually increased to 280°C (15°C/min, held for 10 minutes). Helium (99.999%) served as the carrier gas at a flow rate of 1 ml/min. The injector temperature was set to 240°C. The electron impact ion source, ion trap, and transfer line temperatures were set to 250°C, 220°C, and 280°C, respectively. The mass spectra were recorded at 3 scans/s at 70 eV over the mass range of 50-300 amu.

Transcription of ARHDs genes in strains C1 and WAY2 in the presence of SPMs and biphenyl

To assess the ability of the SPMs to regulate the transcription of the ARHD genes in strains C1 and WAY2, relative transcription of ARHD genes was evaluated at the mRNA level after previous exposure of both strains to SPMs.

Supplementary Figure S2. Relative transcription of *bphA1, nahA1*, and *phtA1* genes in strain C1 after incubation with SPMs and biphenyl for 6h. Samples were prepared in triplicates. The transcription levels were processed by the Common Based Method [13]. Data were normalized to the transcribed levels of the 16S rRNA and *gyrB* genes, analyzed by ANOVA (α = 0.05) followed by the Dunnett post-hoc test and presented with 95% confidence intervals. An asterisk indicates a significant difference in the relative level of gene transcripts compared to the unaffected cells, i.e. control.

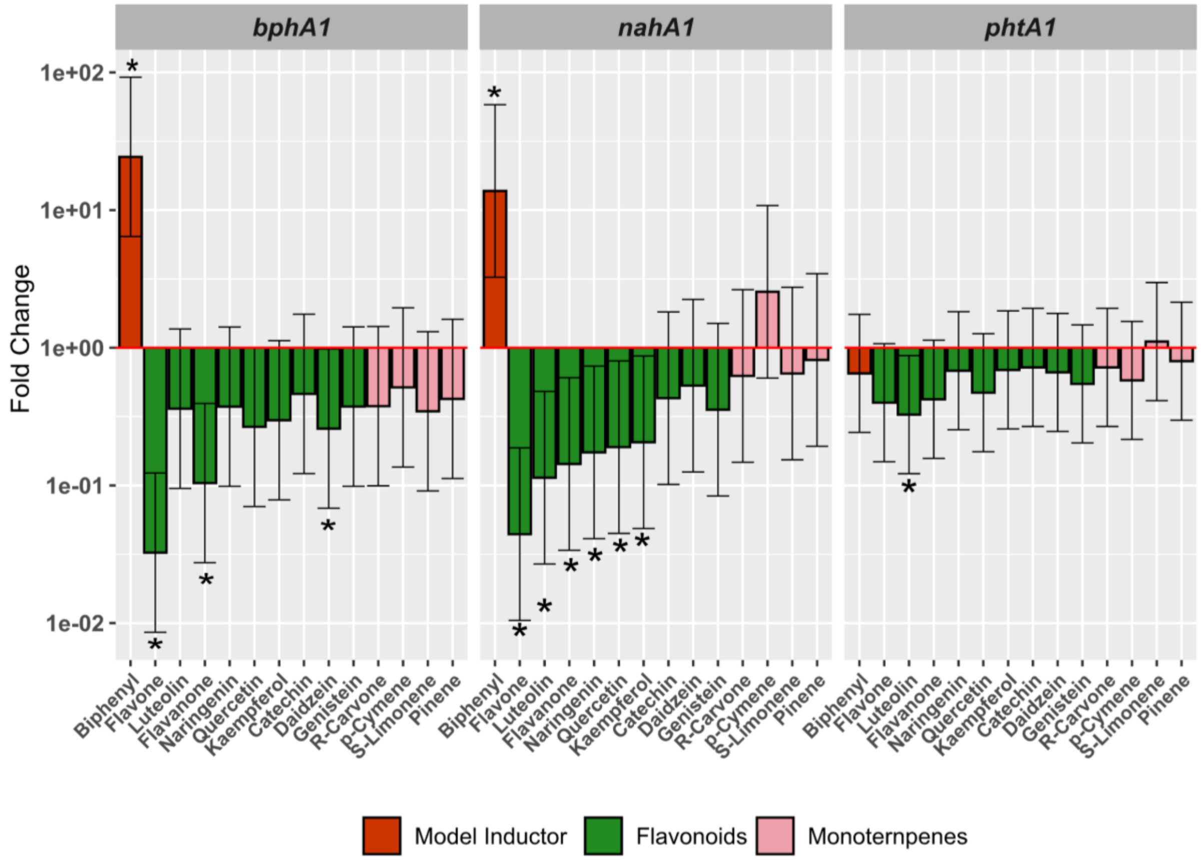

Supplementary Figure S3. Relative transcription of *bphA1, nahA1*, and *phtA1* genes in strain C1 after incubation with *p*-cymene for 6h. Samples were prepared in 7 replicates. The transcription levels were processed by the Common Based Method [13]. Data were normalized to the transcribed levels of the 16S rRNA and *gyrB* genes, analyzed by t-test (α = 0.05), and are presented with 95% confidence intervals. An asterisk indicates a significant difference in the relative level of gene transcripts compared to the unaffected cells, i.e. control.

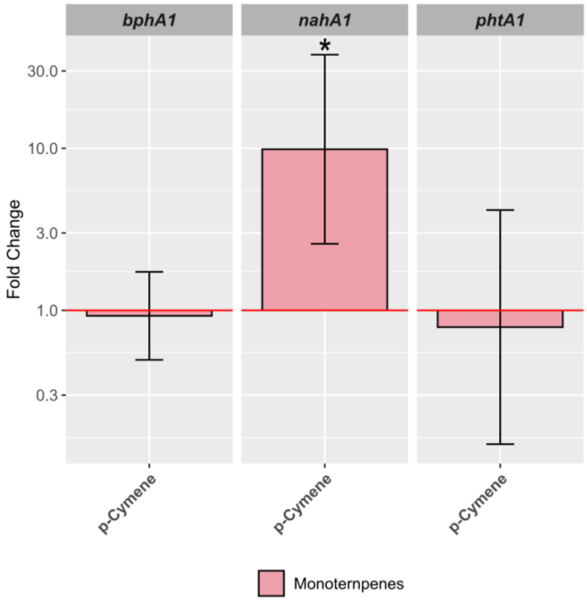

Supplementary Figure S4. Relative transcription of *bphA1a*, *nahA1-bphA1b*, and *etbA1ab* genes in strain WAY2 after incubation with SPMs and biphenyl for 3h. Samples were prepared in triplicates. The transcription levels were processed by the Common Based Method [13]. Data were normalized to the transcribed levels of the 16S rRNA and *gyrB* genes, analyzed by ANOVA (α = 0.05) followed by the Dunnett post-hoc test, and are presented with 95% confidence intervals. An asterisk indicates a significant difference in the relative level of gene transcripts compared to the unaffected cells, i.e. control.

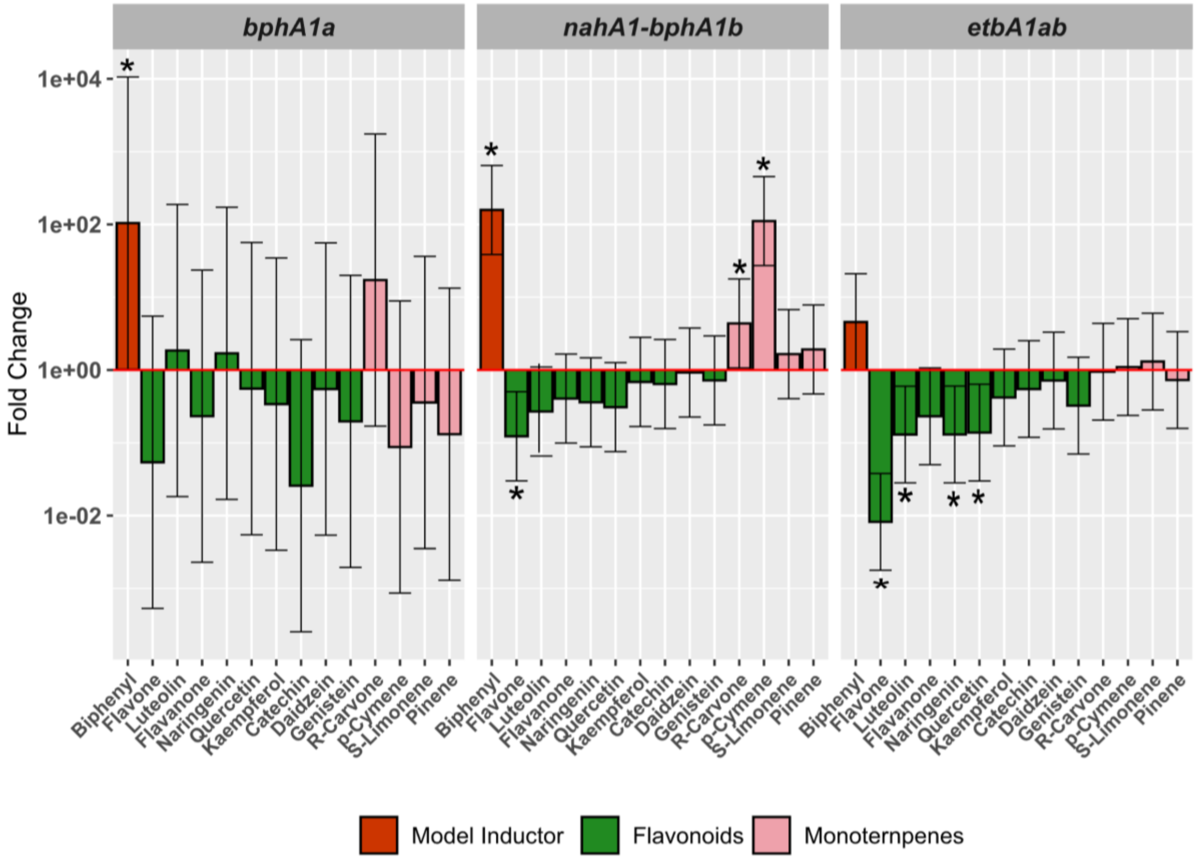

Supplementary Figure S5. Relative transcription of *bphA1a, nahA1-bphA1b,* and *etbA1ab* genes in strain WAY2 after incubation with *R*-carvone and biphenyl for 3h. Samples were prepared in 7 replicates. The transcription levels were processed by the Common Based Method [13]. Data were normalized to the transcribed levels of the 16S rRNA and *gyrB* genes, analyzed by t-test (α = 0.05), and are presented with 95% confidence intervals. An asterisk indicates a significant difference in the relative level of gene transcripts compared to the unaffected cells, i.e. control.

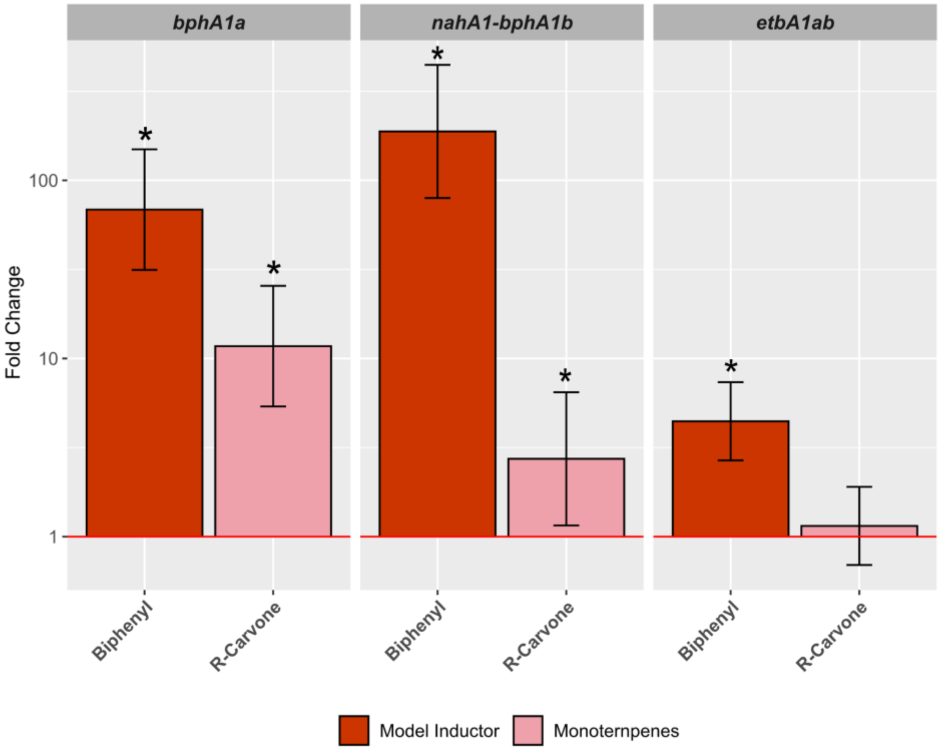

References

1. **Kolmogorov M, Yuan J, Lin Y, Pevzner PA**. Assembly of long, error-prone reads using repeat graphs. *Nat Biotechnol* 2019;37(5):540-546. https://doi.org/10.1038/s41587-019-0072-8
2. **Cumsille A, Durán RE, Rodríguez-Delherbe A, Saona-Urmeneta V, Cámara B et al.** GenoVi, an open-source automated circular genome visualizer for bacteria and archaea. *PLoS Comput Biol* 2023;19(4):e1010998
3. **Parks DH, Imelfort M, Skennerton CT, Hugenholtz P, Tyson GW**. CheckM: assessing the quality of microbial genomes recovered from isolates, single cells, and metagenomes. *Genome Res* 2015;25(7):1043-1055. https://doi.org/10.1101/gr.186072.114
4. **Li W, O'Neill KR, Haft DH, DiCuccio M, Chetvernin V et al.** RefSeq: expanding the prokaryotic genome annotation pipeline reach with protein family model curation. *Nucleic Acids Res* 2021;49(D1):D1020-D1028. https://doi.org/10.1093/nar/gkaa1105
5. **Chaumeil P-A, Mussig AJ, Hugenholtz P, Parks DH**. GTDB-Tk v2: memory friendly classification with the genome taxonomy database. *Bioinformatics* 2022;38(23):5315-5316. https://doi.org/10.1093/bioinformatics/btac672
6. **Potter SC, Luciani A, Eddy SR, Park Y, Lopez R et al.** HMMER web server: 2018 update. *Nucleic acids res.* 2018;46(W1):W200-W204
7. **Garrido-Sanz D, Sansegundo-Lobato P, Redondo-Nieto M, Suman J, Cajthaml T et al.** Analysis of the biodegradative and adaptive potential of the novel polychlorinated biphenyl degrader *Rhodococcus* sp. WAY2 revealed by its complete genome sequence. *Microb Genom* 2020;6(4). https://doi.org/10.1099/mgen.0.000363
8. **Masai E, Yamada A, Healy JM, Hatta T, Kimbara K et al.** Characterization of biphenyl catabolic genes of gram-positive polychlorinated biphenyl degrader *Rhodococcus* sp. strain RHA1. *Appl Environ Microbiol* 1995;61(6):2079-2085. https://doi.org/doi:10.1128/aem.61.6.2079-2085.1995
9. **Anokhina TO, Esikova TZ, Gafarov AB, Polivtseva VN, Baskunov BP et al.** Alternative naphthalene metabolic pathway includes formation of ortho-phthalic acid and cinnamic acid derivatives in the *Rhodococcus opacus* Strain 3D. *Biochemistry (Mosc)* 2020;85(3):355-368. https://doi.org/10.1134/S0006297920030116
10. **Ma B, Lu C, Wang Y, Yu J, Zhao K et al.** A genomic catalogue of soil microbiomes boosts mining of biodiversity and genetic resources. *Nat Commun* 2023;14(1):7318. https://doi.org/10.1038/s41467-023-43000-z
11. **Choi KY, Kim D, Sul WJ, Chae JC, Zylstra GJ et al.** Molecular and biochemical analysis of phthalate and terephthalate degradation by *Rhodococcus* sp. strain DK17. *FEMS Microbiol Lett* 2005;252(2):207-213. https://doi.org/10.1016/j.femsle.2005.08.045
12. **Auffret M, Labbe D, Thouand G, Greer CW, Fayolle-Guichard F**. Degradation of a mixture of hydrocarbons, gasoline, and diesel oil additives by *Rhodococcus aetherivorans* and *Rhodococcus wratislaviensis*. *Appl Environ Microbiol* 2009;75(24):7774-7782. https://doi.org/10.1128/AEM.01117-09
13. **Ganger MT, Dietz GD, Ewing SJ**. A common base method for analysis of qPCR data and the application of simple blocking in qPCR experiments. *BMC bioinformatics* 2017;18(1):1-11. https://doi.org/10.1186/s12859-017-1949-5
